# A Logic‐Memory Transistor with the Integration of Visible Information Sensing‐Memory‐Processing

**DOI:** 10.1002/advs.202002072

**Published:** 2020-09-21

**Authors:** Xiang Hou, Chunsen Liu, Yi Ding, Lan Liu, Shuiyuan Wang, Peng Zhou

**Affiliations:** ^1^ State Key Laboratory of ASIC and System School of Microelectronics Fudan University Shanghai 200433 China; ^2^ School of Computer Science Fudan University Shanghai 200433 China

**Keywords:** logic‐memory transistors, sensing‐memory‐processing, visible information

## Abstract

To meet the demands of future intelligent application scenarios, the time‐efficient information acquisition and energy‐efficient data processing capabilities of terminal electronic systems are indispensable. However, in current commercial visual systems, the visible information is collected by image sensors, converted into digital format data, and transferred to memory units and processors for subsequent processing tasks. As a result, most of the time and energy are wasted in the data conversion and movement, which leads to large time latency and low energy efficiency. Here, based on 2D semiconductor WSe_2_, a logic‐memory transistor that integrates visible information sensing‐memory‐processing capabilities is successfully demonstrated. Furthermore, based on 3 × 3 fabricated devices, an artificial visible information sensing‐memory‐processing system is proposed to perform image distinction tasks, in which the time latency and energy consumption caused by data conversion and movement can be avoided. On the other hand, the logic‐memory transistor can also execute digital logic processing (logic) and logic results storage (memory) at the same time, such as *AND* logic function. Such a logic‐memory transistor could provide a compact approach to develop next‐generation efficient visual systems.

## Graphical abstract

A logic‐memory transistor with the integration of visible information sensing‐memory‐processing is demonstrated. Meaningfully, the logic‐memory transistor can execute logic prosessing and logic results storage at the same time. The logic‐memory transistor also integrates visible information sensing‐memory‐processing capabilities. Furthermore, an artificical visual system is proposed to perform image distinction tasks, in which data conversion and movement can be avoided.

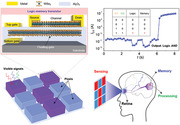

Future intelligent application scenarios (self‐driving cars,^[^ ^1^ ^]^ intelligent robots,^[^ ^2^ ^]^ etc.) will place high demands on the performance of artificial visual systems, such as visible information acquisition without time delay, high energy efficiency and high integration level.^[^ ^3^, ^4^, ^5^ ^]^ Specifically, as the front‐end of artificial visual systems, visual sensors are indispensable for visible information acquisition.^[^ ^6^, ^7^, ^8^, ^9^ ^]^ However, in current commercial technology, the collected visible information cannot be directly processed in its original format, a data conversion process is necessary.^[^ ^10^, ^11^ ^]^ Then, the converted data will be transferred to additional memory units and processing units for subsequent processing tasks, such a complicated data processing flow will bring extra time expenditure and result in large time latency.^[^ ^12^, ^13^, ^14^ ^]^ And the frequent data movement between the separated functional units leads to unacceptable energy consumption,^[^ ^15^, ^16^ ^]^ which contradicts the demands of high energy efficiency and integration level. Recently, massive efforts have been devoted to developing novel artificial visual systems.^[^ ^3^, ^8^, ^9^, ^11^, ^17^, ^18^ ^]^ However, limitations still exist in these works, such as the lack of response capability to visible signals,^[^ ^3^, ^8^ ^]^ the data conversion process is still indispensable^[^ ^9^, ^18^ ^]^ and the data storage is realized by external memory units.^[^ ^11^, ^17^ ^]^ So far, there is no effective solution to completely broken the limitations existing in the commercial artificial visual systems. Therefore, to overcome these predicaments, novel electronic devices that integrate real‐time visible information sensing (without the data conversion process), in situ data memory and processing (remove the frequent data movement) capabilities are urgently needed.

Based on their proper energy band structures and high photoresponsivity,^[^ ^19^, ^20^, ^21^, ^22^ ^]^ 2D semiconductors have great potential for visual information sensing applications.^[^ ^23^, ^24^, ^25^ ^]^ Specifically, with a bandgap ranging from 1.2 to 1.6 eV (depending on the number of stacking layers),^[^ ^26^, ^27^, ^28^ ^]^ the light response of WSe_2_ can cover the entire visible region,^[^ ^29^ ^]^ which makes it a promising building block for future artificial visual systems. In addition, benefiting from their high charge sensitivity and atomic thickness characteristics,^[^ ^30^, ^31^ ^]^ 2D semiconductors show great potential for information storage applications;^[^ ^24^, ^32^, ^33^ ^]^ on the other hand, the high ON/OFF state current ratio and high carrier mobility properties^[^ ^19^, ^34^, ^35^ ^]^ also enable their application prospects in the field of information processing.^[^ ^36^, ^37^ ^]^ Further, a recent work has already proven that 2D semiconductor is a potential solution to integrate information processing and storage in the transistor level, which is difficult to achieve with traditional materials.^[^ ^38^ ^]^ In summary, the rich features of 2D semiconductors could provide a potential path to develop electronic devices that integrate information sensing, memory and processing capabilities.

In this article, based on 2D semiconductor WSe_2_, we design and demonstrate a photoactive logic‐memory transistor with the integration of visible information sensing‐memory‐processing capabilities. First, the constructed logic‐memory transistor exhibits non‐volatile sensing capabilities to visible signals with different wavelengths (red, green, and blue laser signals). And the further realization of optical modulated synaptic plasticity, such as excitatory and long‐term potentiation (LTP) synaptic behavior, enables the real‐time and in situ visible information sensing‐memory‐processing capabilities of the logic‐memory transistor. Based on 3 × 3 fabricated transistors, we propose an artificial visible information sensing‐memory‐processing system to implement image distinction tasks, in which data conversion and data movement are avoided. On the other hand, the logic‐memory transistor can also perform stable *AND* logic processing (logic) and store logic results in situ (memory), and the power consumption of the entire logic cycle is just around 40 fJ at the working frequency of 5 MHz.

**Figure** **1**a shows the 3D schematic of the designed logic‐memory transistor based on a floating‐gate transistor with two gate terminals. The WSe_2_ floating‐gate buried in the Al_2_O_3_ dielectric can be regulated by the bottom and top gate terminals. In purpose, the entire WSe_2_ channel of the floating‐gate transistor is completely overlapped by part of the WSe_2_ floating‐gate, which is important for the implementation of subsequent logic‐memory and visible signal sensing‐memory‐processing capabilities (see more device fabrication and structure details in Section S1 in the Supporting Information). Figure 1b is the false‐colored scanning electron microscope (SEM) image of the logic‐memory transistor, the red and blue regions represent floating‐gate and channel respectively.

**Figure 1:**
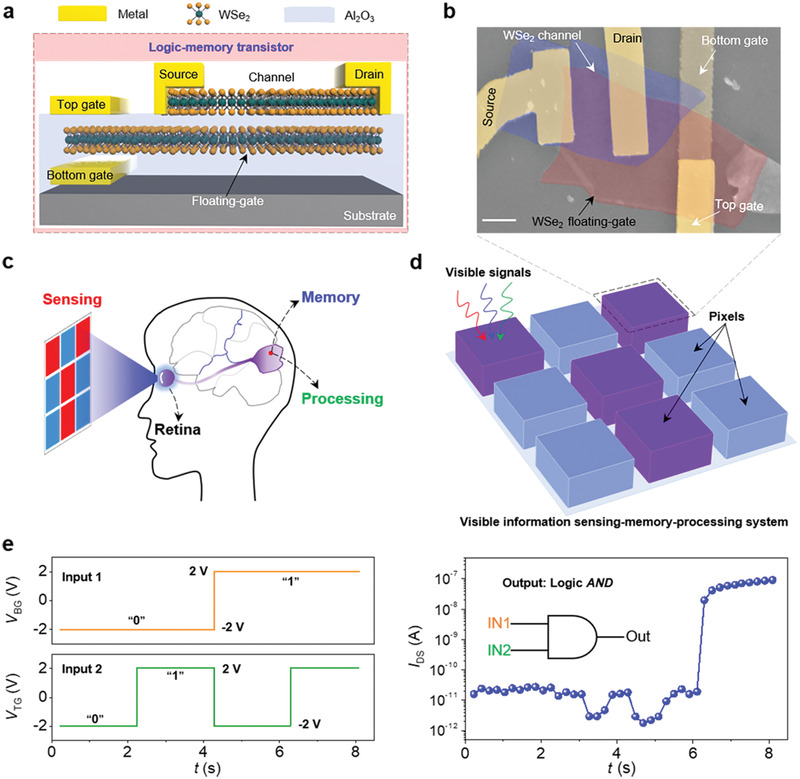
a) 3D schematic view of the logic‐memory transistor, in which 2D WSe_2_ serves as the floating‐gate and channel, Al_2_O_3_ is the top/bottom gate dielectric. b) False‐colored SEM image of the fabricated logic‐memory transistor in top view, the scale bar is 2 µm. c) Simplified diagram of the human visual system. d) A diagram of the visible information sensing‐memory‐processing system composed of 3 × 3 logic‐memory transistors. e) Demonstration of the *AND* logic function, the left two panels are input signals and the right panel is the logic output signals. During the measurement of output signals, *V*_DS_ is fixed at 1 V. d) Robustness performance of the demonstrated *AND* logic gate.

Figure 1c is a simplified diagram of the human visual system, which is capable of visible information sensing‐memory‐processing. As the front‐end, the retina can directly respond to the visible signals.^[^ ^39^, ^40^ ^]^ Then, the sensory visual data will be delivered to the visual cortex of the brain through the optic nerve for subsequent memory and processing.^[^ ^7^, ^41^ ^]^ A step further, we demonstrated a visible information sensing‐memory‐processing system (as shown in Figure 1d) to mimic the human visual system, in which one logic‐memory transistor corresponds to one image pixel.

In conventional electronic system, the data processing (in processor unit) and data storage (in memory unit) are physically separated. During the processing tasks, there is a frequent data movement between the processor and memory unit, which results in large time latency and low energy efficiency.^[^ ^16^, ^42^ ^]^ As a basic logic gate, *AND* gate is an essential component of the processor unit, and other logic gates can be transformed from *AND* gate in a certain form.^[^ ^38^ ^]^ Here, for the first time, we demonstrate a logic‐memory transistor that can do both *AND* logic processing (logic) and store logic results in situ (memory) at the same time, which has great application potential for avoiding the data movement in the future electronic system.

During the measurement of the *AND* logic gate, bottom voltage bias (*V*_BG_) and top voltage bias (*V*_TG_) serve as the input signals (defined as IN1 and IN2, respectively), and the channel current of the logic‐memory transistor (*I*_DS_) is the logic output signal (the input and output signals are all electrical signals). Specifically, for the input signals, a negative voltage bias (such as −2 V) is defined as logic “0” and a positive voltage bias (such as 2 V) is defined as logic “1.” Figure 1e presents the output signals of the logic‐memory transistor to four different combinations of input signals. In the case where *V*_BG_ = −2 V and *V*_TG_ = −2 V (input IN‐00), the output current is around 10^−11^ A, corresponding to logic “0.” In the same way, when inputting IN‐01 or IN‐10, the output current is also around 10^−11^ A. Interestingly, when *V*_BG_ = *V*_TG_ = 2 V (input IN‐11), the output current turns into a high value (≈10^−7^ A) which corresponds to logic “1.” Therefore, *AND* logic gate is successfully implemented on the single logic‐memory transistor. To study the robustness performance of the demonstrated *AND* logic gate, periodic voltage input signals were applied on the bottom and top gate terminals. As illustrated in Figure S3 in the Supporting Information, the output signals indicate that the *AND* logic function is still stable after 100 logic operation cycles. In addition, we made a systematical exploration on the impact of device parameters on logic behavior (more details are presented in Section S3 in the Supporting Information). Measurement results indicate that the fluctuation of channel thickness does not affect the logic behavior, on the other hand, the operating voltage of the logic gate gradually increases as the dielectric gets thicker.

Then, the high‐frequency property of the *AND* logic gate is investigated. In the case of the input signal frequency of 5 MHz, the *AND* logic function is still workable (see Section S4 in the Supporting Information). At this operating frequency, the static power consumption of the logic gate during a logic cycle (IN‐00, IN‐01, IN‐10, IN‐11, voltage amplitude of 2 V) is calculated to be just around 40 fJ, the detailed calculation process of the power consumption is presented in Section S4 in the Supporting Information. Moreover, benefitting from the enhancement type of WSe_2_ channel, the subthreshold (no gate voltage bias, *V*_BG_ = *V*_TG_ = 0 V) leakage current (*I*_SUB_) of the logic‐memory transistor is around 10^−11^ A (Figure S8, Supporting Information), which means that the standby power consumption of the demonstrated logic gate can be extremely low.

Besides the implementation of *AND* logic gate, the constructed logic‐memory transistor can also accomplish the logic results storage during the logic processing in the same single cell. Here, we show more details. During logic‐00 operation, the channel current is around 10^−11^ A, corresponding to logic output “0.” When the input voltage signals are revoked, the WSe_2_ channel remains in low current (OFF) state (matched with the logic‐00 processing result, as shown in **Figure** **2**a). The same as logic‐00 operation, the results of logic‐01 and logic‐10 operations can also be stored in the transistor during the logic processing, the detailed data are shown in Figure 2b,c. On the other hand, in the case of *V*_BG_ = 2 V and *V*_TG_ = 2 V (logic‐11 operation), the output current switches into a high value (Figure 2d). When the voltage input signals return to 0 V, the WSe_2_ channel does not turn into the OFF state, instead, it maintains a relatively high current value (≈10^−9^ A), which has a ratio of 100 times compared with the OFF‐state current. As a result, after the logic‐11 operation, the logic result can also be stored in the transistor. The schematic diagram presented in Figure 2e clearly demonstrates the in situ logic results storage capability of the *AND* logic gate. Figure 2f is the truth table of the *AND* logic gate with in situ logic results storage capability, only under the condition that IN1 = IN2 = “1,” both the logic result and stored result are “1.” In summary, the logic results can be in situ stored in the logic‐memory transistor during logic processing operations, and no additional memory cells and memory operations are needed. The detailed operation mechanism of the logic gate with in situ results storage capability is presented in Section S7 in the Supporting Information. Furthermore, it should be pointed out that the relative positional relationship between the gate electrodes does not affect the realization of logic‐memory functionalities, see more details in Figure S14 in the Supporting Information.

**Figure 2:**
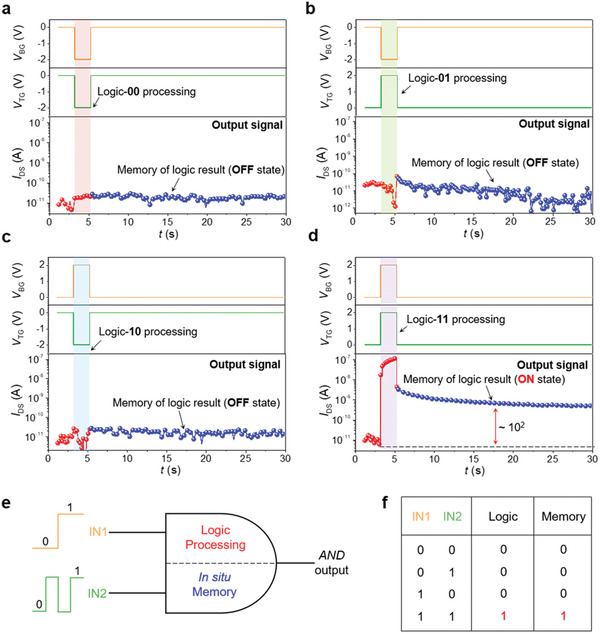
In situ logic results storage capability of the demonstrated *AND* logic gate. a–d) Output signal monitoring during the logic processing operations (IN‐00, IN‐01, IN‐10, and IN‐11) and after the logic processing operations. *V*_DS_ is fixed at 1 V during the monitoring process. e) A schematic diagram of the *AND* logic gate to describe the in situ logic result storage capability during the logic processing operations. f) Summary table of the logic processing output and the stored output under different input signals.

For artificial visual systems, visible signal sensing capability is the precondition of subsequent tasks, such as image memorization and distinction. Owing to the appropriate energy band structure of 2D WSe_2_,^[^ ^26^, ^27^ ^]^ the demonstrated logic‐memory transistor also exhibits photoactive characteristics. Specifically, when exposed to a laser‐irradiated environment, the logic‐memory transistor exhibits a noticeable photoresponse. In a dark environment, the WSe_2_ channel is at an initial state, then, *I*_DS_ shows a sudden increment when blue laser pulse (wavelength is 473 nm) is applied to the logic‐memory transistor (as shown in **Figure** **3**a).

**Figure 3:**
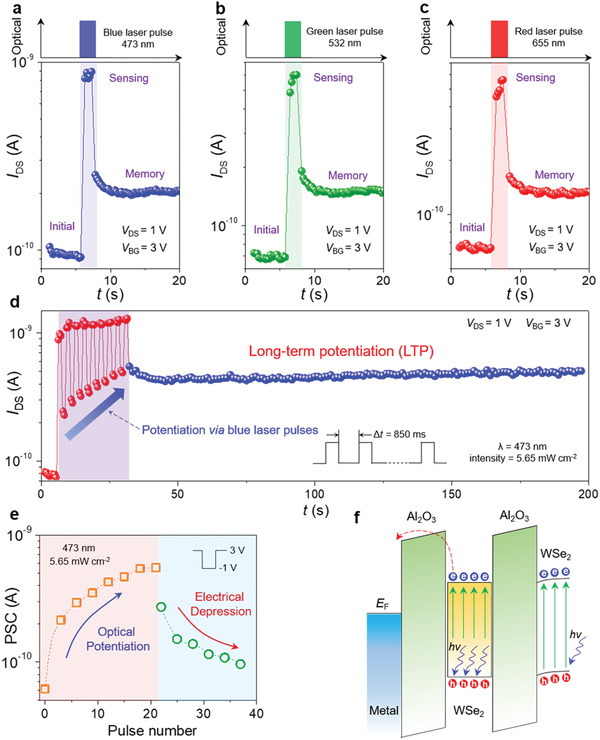
Non‐volatile visible signals sensing capability and optical modulated synaptic plasticity. a–c) The channel current monitoring during the different laser signals (blue, green, and red) sensing and memory processes. *V*_DS_ is fixed at 1 V and bottom gate voltage bias is 3 V, the width of the laser pulses is about 950 ms. d) LTP synaptic characteristics of the logic‐memory transistor. The wavelength of the laser training signals is 473 nm, the intensity is 5.65 mW cm^−2^, and the interval is 850 ms. e) Extracted post‐synaptic current (PSC) during the optical potentiation and electrical depression processes. f) An energy band diagram of the logic‐memory transistor under the excitation of laser and a bottom gate voltage bias.

More importantly, after the laser irradiation, instead of returning to the initial state, *I*_DS_ remains at a relatively high value, which indicates that the sensory data can be in situ stored without conversion and movement processes. Besides the blue laser signal, the photoactive logic‐memory transistor also exhibits non‐volatile sensing capability to green (wavelength is 532 nm) and red (wavelength is 655 nm) laser signals, as presented in Figure 3b,c. As the wavelength of the laser signal decreases, the responsivity of the logic‐memory transistor shows a gradual increase (the ratio between photocurrent and dark current increases gradually, see Figure 3a–c; the more optoelectrical properties of the logic‐memory transistor are presented in Section S8 in the Supporting Information).

To break the limitations of conventional electronic systems, artificial synaptic devices that integrate data memory and processing is an ideal solution.^[^ ^43^, ^44^ ^]^ Here, based on the visible signal sensing capability, optical modulated synaptic plasticity is successfully demonstrated. As illustrated in Figure 3d, under the stimulation of intermittent laser signals (blue laser pulses, 850 ms interval), *I*_DS_ gradually increases, which exhibits an optical potentiation property. After the potentiation of intermittent laser signals, *I*_DS_ shows a non‐volatile behavior (as shown in Figure 3d, during the monitoring time of more than 160 s, *I*_DS_ remains stable), therefore, optical modulated long‐term potentiation (LTP) is realized on the photoactive logic‐memory transistor.

More than excitatory synaptic plasticity, long‐term depression (LTD) synaptic plasticity can also be achieved by applying electrical signals (3 V bias, −1 V peak, duration of 850 ms, interval of 850 ms) to the bottom gate terminal. The extracted post‐synaptic current (PSC) from the excitatory and depressible plasticity modulation processes are plotted in Figure 3e, which clearly demonstrates the optical potentiation and electrical depression. PSC shows an obvious step‐by‐step rise as the number of laser pulses increases from 0 to 20. Conversely, PSC gradually returns to the initial state under the action of voltage pulses. In addition, other synaptic behaviors, such as paired‐pulse facilitation (PPF) and spike‐timing dependent plasticity (STDP), are implemented on the logic‐memory transistor (see more details in Section S8 in the Supporting Information).

In summary, the non‐volatile visible signal sensing capability and optical modulated synaptic behaviors enable the demonstrated logic‐memory transistor to perform real‐time and in situ human visible signals (composed of red light, green light, and blue light in different proportions) sensing‐memory‐processing, which has good application prospects for developing efficient artificial visual system.

We draw an energy band diagram of the logic‐memory transistor under the excitation of laser to explain the mechanism of the non‐volatile visible signal sensing capability, as shown in Figure 3f. Under laser irradiation, the incident photons with an energy exceeding the bandgap of WSe_2_ can generate electron‐hole pairs in the channel and the floating‐gate.^[^ ^45^ ^]^ As a result, the generated channel photocurrent leads to a large increase in *I*_DS_. Especially, during the laser irradiation process, the laser‐generated electrons can tunnel from the conduction band of the WSe_2_ floating‐gate into the bottom gate terminal under a positive bottom gate voltage bias (*V*_BG_). Then, after the laser irradiation, the generated electrons in the conduction band will recombine with the holes in the valence band. In the floating‐gate, due to the absence of some electrons (tunneling into the bottom gate terminal), there will be a certain number of unpaired holes. The unpaired holes can remain in the floating‐gate after the laser irradiation and enhance the WSe_2_ channel, which maintains *I*_DS_ at a relatively high value.

Here, for the first time, we propose an artificial visible information sensing‐memory‐processing system based on 3 × 3 fabricated logic‐memory transistors, in which data conversion and data movement are no longer needed (the optical images of the 3 × 3 fabricated transistors are presented in Section S9 in the Supporting Information). Specifically, the proposed system can perform real‐time image acquisition, in situ image memorization and image distinction tasks.

**Figure** **4**a presents the images of the letters with 3 × 3 pixels, which will be used for subsequent tasks. A detailed process flow of the proposed artificial visible information sensing‐memory‐processing system is illustrated in Figure . First, in the initial state, all the logic‐memory transistors are in a low conductance state. Benefitting from the optical modulated LTP synaptic plasticity of the logic‐memory transistors, under the continuous training of laser signals, the system can realize real‐time image acquisition and in situ memorization. For example, as shown in Figure 4c, the evolution of the conductance maps (one logic‐memory transistor corresponds to one pixel) clearly presents the acquisition and memorization processes of the letter “Y” image. Under the training of laser signals (wavelength is 473 nm, intensity is 5.65 mW cm^−2^, duration of 850 ms), the conductance of the selected logic‐memory transistors will gradually increase. As a result, after 10 sessions of laser signal training, there is an obvious conductance difference between the selected and unselected logic‐memory transistors, which forms the image of letter “Y.” The conductance map after 100 s demonstrates the in situ memorization of sensory letter “Y” image.

**Figure 4:**
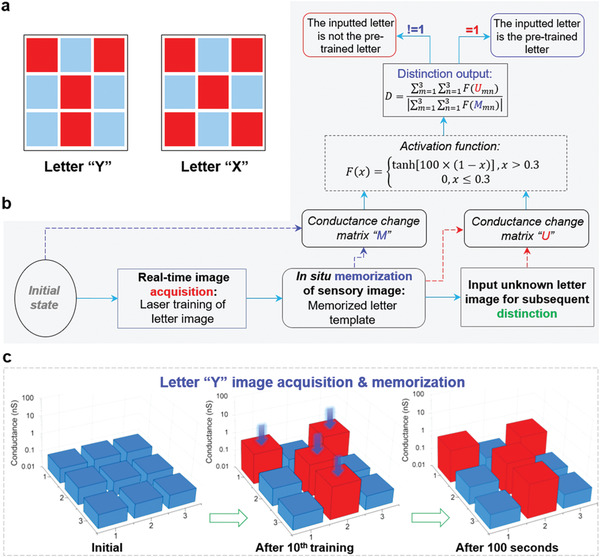
Artificial visible information sensing‐memory‐processing system based on 3 × 3 logic‐memory transistors. a) Images of the letters with 3 × 3 pixels, which will be used for subsequent tasks. b) The process flow of the proposed artificial visible information sensing‐memory‐processing system. c) The conductance evolution of the 3 × 3 logic‐memory transistors during the process of the letter “Y” image acquisition and memorization.

To perform image distinction tasks, an image of a reference letter is inputted into the system at first (by applying the corresponding laser signals to the 3 × 3 logic‐memory transistors in the initial state), as a result, the conductance of the logic‐memory transistors will be updated and in situ memorized. By measuring the conductance of the 3 × 3 logic‐memory transistors before and after the reference letter image training, we can get a *3* × *3* conductance change matrix *M*. Subsequently, laser training signals corresponding to an unknown letter image is applied to the pre‐trained template, and the conductance of the 3 × 3 logic‐memory transistors will be updated again. In the same way, we can also get a *3* × *3* matrix *U* by measuring the conductance change.

Based on the conductance change matrix, *M* and *U*, the distinction between the images of the reference letter and unknown letter can be performed. Specifically, the rule of distinction process is defined as

(1) $$D=\frac{\sum_{m=1}^{3}\sum_{n=1}^{3}F\left(U_{mn}\right)}{\left|\sum_{m=1}^{3}\sum_{n=1}^{3}F\left(M_{mn}\right)\right|}$$

where *D* is the distinction output, *U_mn_* is the element of matrix *U* (1≤ *m*, *n* ≤3), *M_mn_* is the element of matrix *M* (1≤ *m*, *n* ≤3), and *F* is an activation function. Only in the condition that *D* equals to 1, the inputted unknown letter matches with the reference letter; otherwise, the inputted unknown letter does not match with the reference letter. The expression of activation function *F* is

(2) $$F\left(x\right)=\left\{\begin{array}{cc} \tanh[100\times (1-x)], & x>0.3\\ 0, & x\leq 0.3 \end{array}\right)$$

In **Figure** **5**a, we apply the proposed artificial visible information sensing‐memory‐processing system to perform a distinction between the letter “Y” image and the unknown letter 1 (“X”) image. Specifically, laser training signals corresponding to letter “Y” image are applied to the 3 × 3 logic‐memory transistors that in the initial state, and the conductance change matrix *M*_1_ is further calculated (see more details in Section S10 in the Supporting Information). Then, the image of the unknown letter 1 (“X”) is inputted into the system, according to the conductance measurement results, we can get the conductance change matrix *U*_1_ (see more details in Section S10 in the Supporting Information). Based on *M*_1_, *U*_1_ and the distinction rule, the distinction output is calculated to be 0.25, not equal to 1, which indicates that the inputted unknown letter 1 (“X”) is not the reference letter “Y.” After the distinction process between the letter “Y” image and unknown letter 1 (“X”), the 3 × 3 logic‐memory transistors are refreshed back to the initial state. Figure 5b presents the distinction between the letter “Y” image and the unknown letter 2 (“Y”) image. The laser training signals corresponding to letter “Y” image and unknown letter 2 (“Y”) image are applied to the 3 × 3 logic‐memory transistors successively, and the conductance change matrix *M*_2_ and *U*_2_ are calculated (see more details in Section S10 in the Supporting Information). As shown in Figure 5b, the calculated distinction output is equal to 1, which means that the inputted unknown letter 2 (“Y”) is letter “Y.”

**Figure 5:**
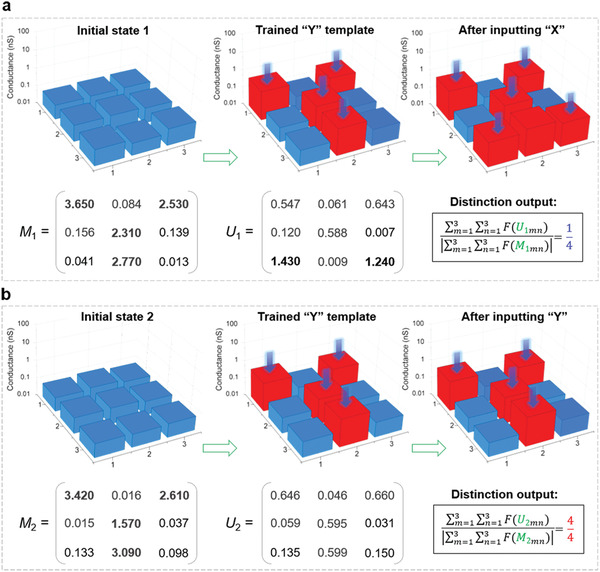
Image distinction via the proposed artificial visible information sensing‐memory‐processing system. a) The distinction between letter “Y” image and the image of unknown letter 1 (“X”), the conductance maps of the 3 × 3 logic‐memory transistors at 3 different stages (initial state 1, after the training of letter “Y” image and after inputting the imageof unknown letter 1) are illustrated. b) The distinction between the letter “Y” image and the image of unknown letter 2 (“Y”).

In this work, by unique structural design and taking advantage of 2D semiconductor WSe_2_ with photoresponse, we proposed a photoactive logic‐memory transistor. The logic‐memory transistor can perform stable *AND* logic processing, and the power consumption of the entire logic cycle is just around 40 fJ at a working frequency of 5 MHz. Meaningfully, the logic results can be in situ stored in the logic‐memory transistor during the logic processing, which has the potential to remove the data movement between processor and memory units. Moreover, based on the non‐volatile visible signal sensing capability and optical modulated synaptic plasticity, the integration of visible information sensing‐memory‐processing is successfully implemented on the logic‐memory transistor. Finally, we demonstrated an artificial visible information sensing‐memory‐processing system based on 3 × 3 logic‐memory transistors to execute real‐time image acquisition, in situ image memorization and distinction tasks, in which the data conversion and movement are no longer needed.

## Experimental Section

##### Device Fabrication

First, the pattern of the bottom gate electrode was defined by electron beam lithography (EBL) technology using polymethyl methacrylate (PMMA) (AR‐679.04) polymer, then, the Cr/Au (5 nm/15 nm) bottom electrode was deposited by e‐beam evaporation (EBE). After metal deposition, the entire sample was immersed in acetone to remove the residual metal and polymer. Having completed the bottom gate electrode deposition, 9‐nm thick gate dielectric (Al_2_O_3_) was deposited via atomic layer deposition (ALD). During the ALD process, the reacting temperature was set as 300 °C, water and trimethylaluminum (TMA) source were alternatively pumped into the chamber. The layered WSe_2_ floating‐gate and channel were exfoliated from bulk WSe_2_. Then, the WSe_2_ floating‐gate was transferred onto the sample using water‐soluble polyvinyl alcohol (PVA) as the carrier via wet transfer technology. Before the deposition of the top gate dielectric, a 1‐nm thick Y seeding layer was deposited on the floating‐gate. Next, the top gate dielectric was deposited on the sample via ALD, and the WSe_2_ channel was aligned with the floating‐gate by using the transfer platform. Finally, the deposition process of the top gate, drain and source electrodes was the same as for the bottom electrodes. (the specific device fabrication flow is illustrated in Figure S1 in the Supporting Information)

##### Characterizations

The optical measurements were performed on the TTL/analog modulated multi‐wavelengths (473, 532, and 655 nm) laser system. The electrical properties of the fabricated logic‐memory transistor were measured by the Keithley 4200‐SCS semiconductor analyzer. During the *NAND* logic function test, the pull‐up resistor was connected with the measurement system via a resistance box. The current/voltage signal conversion in the high‐frequency test was realized on a FEMTO DHPCA‐100 variable gain high‐speed current amplifier. The thickness of the WSe_2_ channel was measured by AFM (Bruker Veeco MultiMode 8 system) in tapping mode.

## Conflict of Interest

The authors declare no conflict of interest.

## Supporting information

Supporting Information
