# Supplementary material for: A Logic‐Memory Transistor with the Integration of Visible Information Sensing‐Memory‐Processing
Source: Adv Sci (Weinh). 2020 Sep 21;7(21):2002072. doi: 10.1002/advs.202002072 (PMC7610317; doi:10.1002/advs.202002072)
Supplement: Supplementary file 1 — Supporting Information [file ADVS-7-2002072-s001.pdf]

© 2020 Wiley-VCH GmbH

ADVANCED SCIENCE
Open Access

# Supporting Information

for *Adv. Sci.*, DOI: 10.1002/advs. 202002072

# A Logic-Memory Transistor with the Integration of Visible Information Sensing-Memory-Processing

*Xiang Hou, Chunsen Liu, Yi Ding, Lan Liu, Shuiyuan Wang, and Peng Zhou**

# Supplementary Information for

# A logic-memory transistor with the integration of visible information sensing-memory-processing

*Xiang Hou*[1†], *Chunsen Liu*[1,2†], *Yi Ding*[1], *Lan Liu*[1], *Shuiyuan Wang*[1] *and Peng Zhou*[1]*

[1]State Key Laboratory of ASIC and System, School of Microelectronics, Fudan University, Shanghai 200433, China

[2]School of Computer Science, Fudan University, Shanghai 200433, China

[†]These authors contributed equally to this work.

*E-mail: pengzhou@fudan.edu.cn

# Table of contents

1. Fabrication and characterization of the logic-memory transistor ....................3

2. Robustness performance of the demonstrated *AND* logic gate........................5

3. Device parameter dependence of the demonstrated *AND* logic gate ...............6

4. High-frequency and low-power consumption properties ................................9

5. Implementation of *NAND* logic gate.............................................................12

6. Dynamic logic processing & *in situ* results storage properties......................14

7. Mechanism of *in situ* results storage capabilities .........................................15

8. Optoelectrical and synaptic properties of the logic-memory transistor .........18

9. The optical images of the 3×3 logic-memory transistors...............................22

10. Calculation of the conductance change matrices .......................................23

References........................................................................................................27

## 1. Fabrication and characterization of the logic-memory transistor

We draw a flow diagram to clearly demonstrate the device fabrication process of the logic-memory transistor, as illustrated in Supplementary Figure 1.

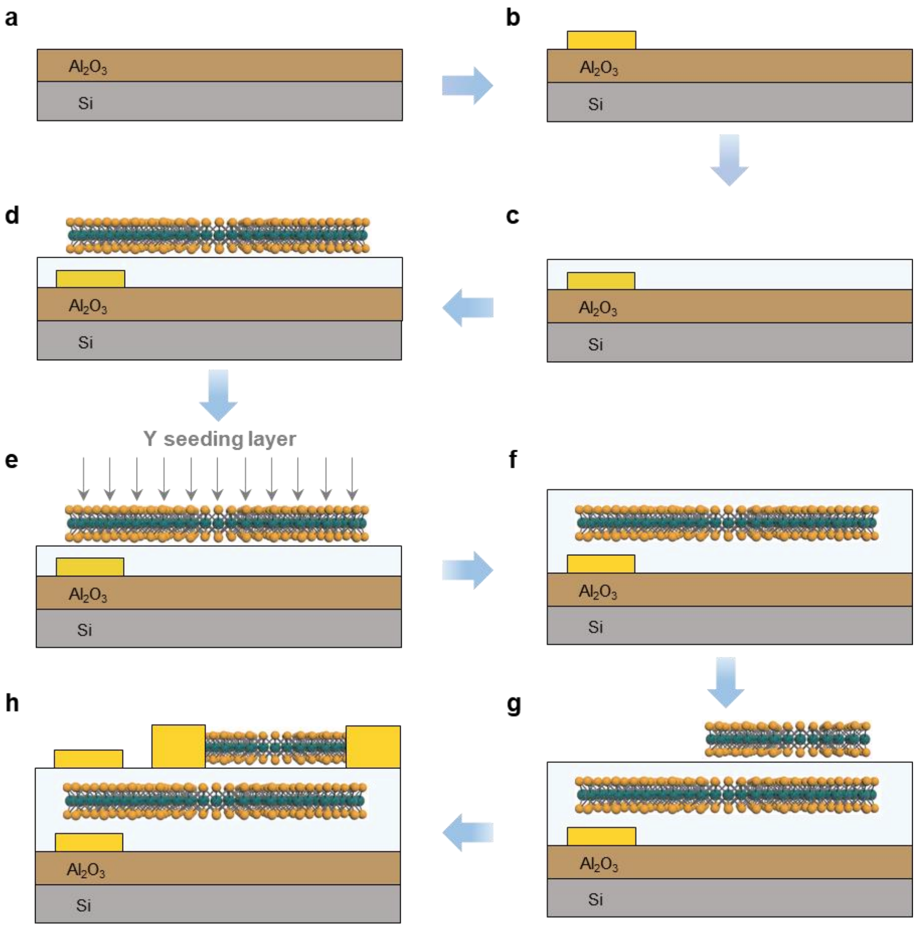


**Supplementary Figure 1.** Schematic of the logic-memory transistor fabrication flow. **a**, Substrate preparation: 30-nm thick $Al_2O_3$ dielectric was deposited on the heavily-doped silicon substrate, then metal markers (provide alignment base for the subsequent EBL processes) were patterned on the substrate *via* ultraviolet lithography technology. **b**, Bottom gate electrode deposition: the electrode pattern was defined by EBL and Cr/Au metal was deposited by EBE. **c**, Bottom gate dielectric growth by ALD. **d**, $WSe_2$ floating-gate transferred onto the substrate. **e**, Deposition of the 1-nm Y seeding layer on the $WSe_2$ floating-gate, which can enhance the quality of the

subsequent top gate dielectric deposition. **f**, Deposition of the top gate dielectric *via* ALD. **g**, Transfer of the $WSe_2$ channel onto the sample. **h**, Top gate, source and drain metal electrodes were deposited as same as the processes of bottom gate electrode.

To further demonstrate the device structure and material composition, we provide the Raman characterization of the logic-memory transistor based on a floating-gate transistor. When the excitation wavelength is around 249.364 $cm^{-1}$, the $WSe_2$ floating-gate and channel exhibit strong intensity, as shown in Supplementary Figure 2a. The extracted Raman spectrum (Supplementary Figure 2b) of floating-gate and channel shows a higher peak near 249 $cm^{-1}$ and a lower peak near 310 $cm^{-1}$, corresponding to the in-plane vibration model ($A_{1g}$) and vertical vibration model ($B^1_{2g}$) of $WSe_2$,[1,2] respectively.

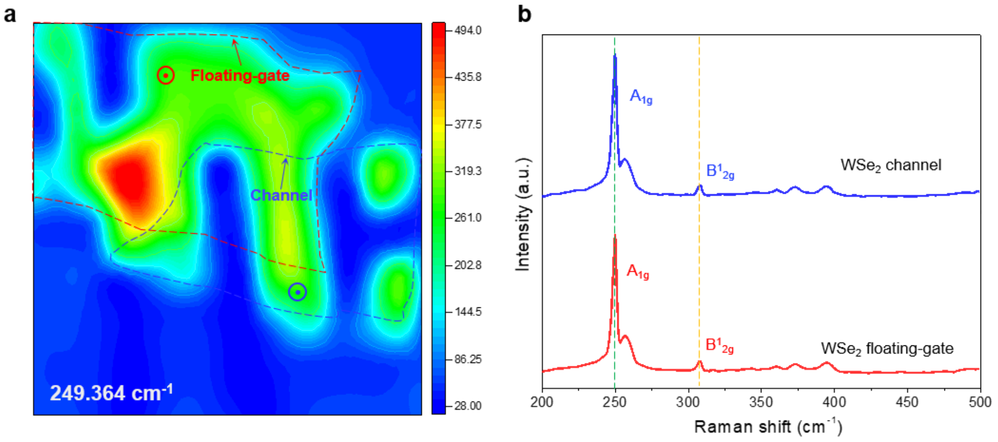


**Supplementary Figure 2.** Raman characterizations of the logic-memory transistor based on a floating-gate transistor. **a**, Raman map at the peak of 249.364 $cm^{-1}$, floating-gate part and channel part are highlighted by the red dashed line and blue

dashed line respectively. **b**, Raman spectra of $WSe_2$ floating-gate and channel, the wavelength of the excitation laser is 532 nm.

## 2. Robustness performance of the demonstrated *AND* logic gate

To further study the robustness performance of the demonstrated *AND* logic gate, we measured the device repeatedly. As illustrated in Supplementary Figure 3, the logic gate can still execute *AND* logic processing after 100 logic operation cycles.

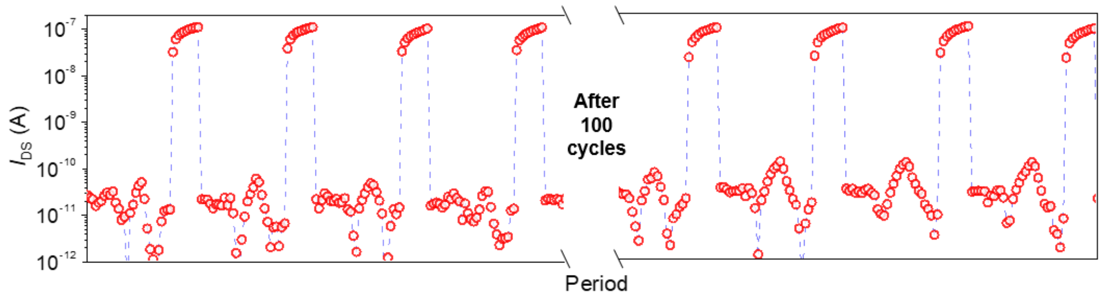


**Supplementary Figure 3.** Robustness performance of the *AND* logic gate. During the measurement of output signals, $V_{DS}$ is fixed at 1 V.

## 3. Device parameter dependence of the demonstrated *AND* logic gate

To further explore the dependence between logic behavior and channel thickness, we fabricated logic-memory transistors with various channel thicknesses (the thickness of the bottom/top gate dielectric was fixed at 9 nm). The channel thickness of the fabricated devices was confirmed by atomic force microscope (AFM), the extracted height images indicate that the thickness is around 3 nm, 5 nm, 7 nm, 9 nm and 11 nm respectively (see Supplementary Figure 4a~e). As summarized in Supplementary Figure 4f, all the fabricated logic-memory transistors exhibit stable *AND* logic function at 2 V or 3 V operating voltage.

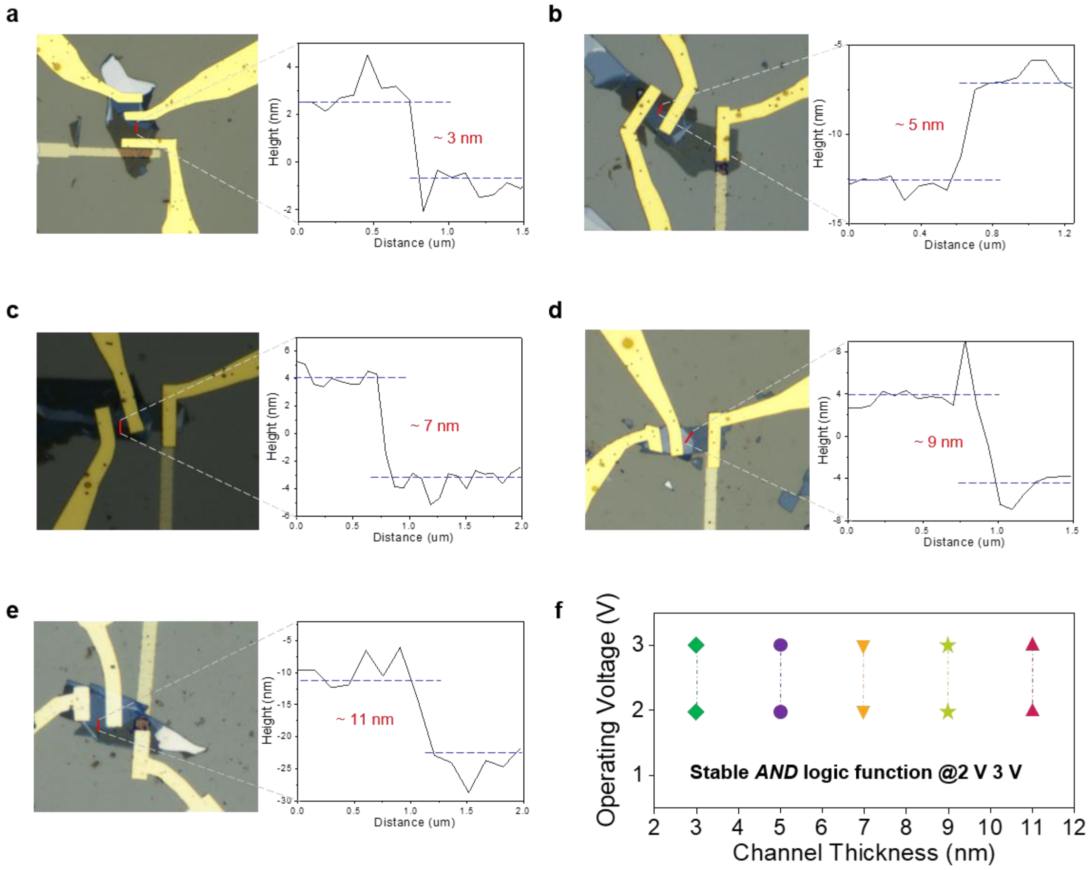


**Supplementary Figure 4.** Channel thickness dependence of the *AND* logic gate. **a~e**, Optical images and AFM height images of the fabricated logic-memory transistors.

The red cut lines in the optical images represent the position of height difference measurement. Thickness of channel is calculated from the height difference between the blue dashed lines. **f**, A logic operating voltage summarization of the *AND* logic gates with different channel thicknesses.

Based on the conclusion that the channel thickness variation cannot directly affect the logic behavior of the *AND* logic gate, we further explored the gate dielectric thickness dependence of logic behavior. The measurement results indicate that the logic-memory transistor with 7-nm thick dielectric can realize *AND* logic function under 1 V and 2 V operating voltage, as shown in Supplementary Figure 5a~b; when the thickness of the gate dielectric increases to 9 nm, logic function can be realized under 2 V and 3 V operating voltage (Supplementary Figure 5c~d); in the case of the thickness of gate dielectric is 11 nm, the logic-memory transistor can perform *AND* logic function under 3 V, 4 V and 5 V operating voltage (Supplementary Figure 5e~g). An operating voltage change trend chart is given in Supplementary Figure 5h, it's obvious that the operating voltage of the *AND* logic gate increases as the dielectric gets thicker. This phenomenon can be explained as follows: thickening of the gate dielectric will reduce the electric field strength generated by the bottom/top gate, which weakens the regulation ability of the bottom/top gate.[3] Therefore, the operating voltage of *AND* logic gate gets higher with the increase of the gate dielectric thickness.

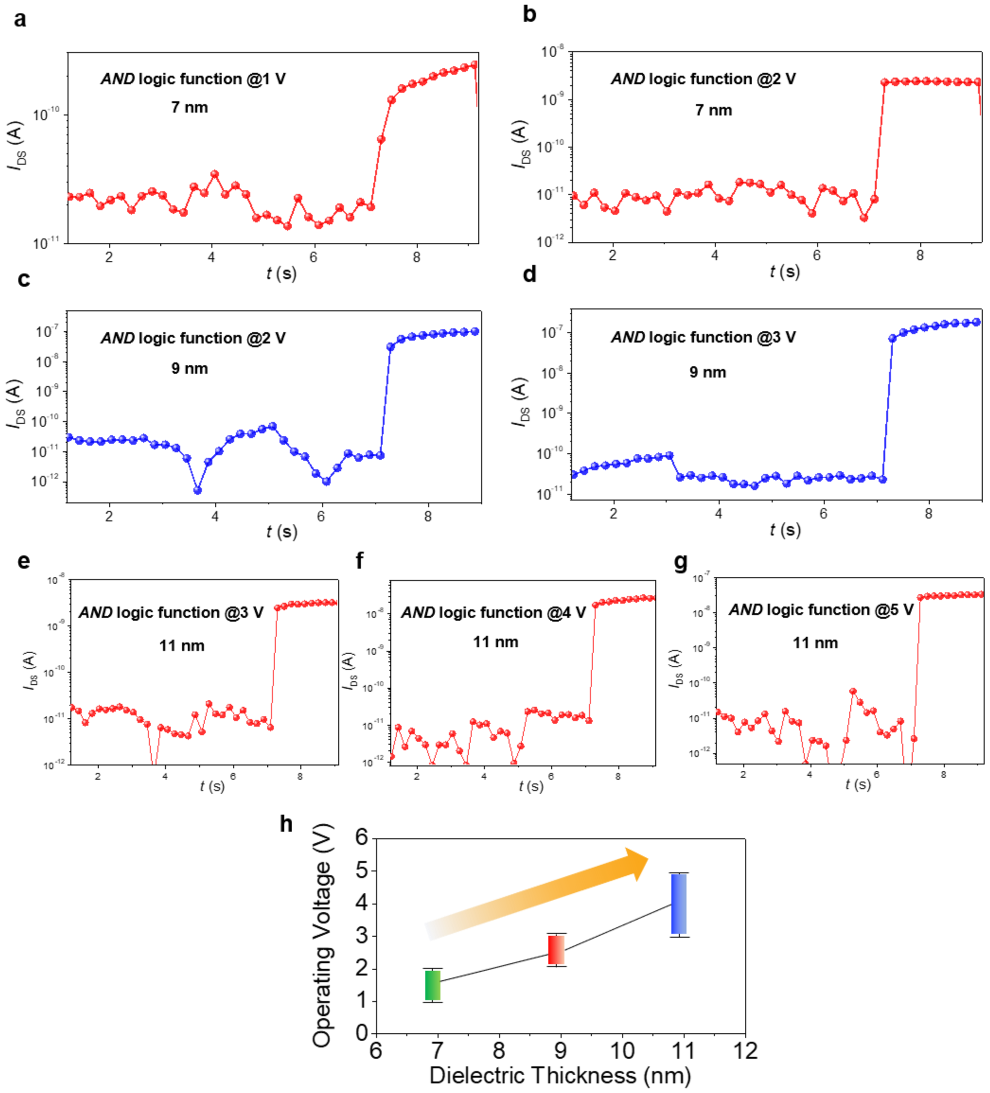


**Supplementary Figure 5.** Logic operating voltage range of logic-memory transistors with different dielectric thicknesses. **a**,**b**, Logic output signal of the logic-memory transistor with 7-nm thick gate dielectric under 1 V and 2 V operating voltage respectively. **c**,**d**, Logic output signal of the logic-memory transistor with 9-nm thick gate dielectric under 2 V and 3 V operating voltage respectively. **e**~**g**, Logic output signal of the logic-memory transistor with 11-nm thick gate dielectric under 3 V, 4 V and 5 V operating voltage respectively. **h**, Operating voltage change trend chart of the logic-memory transistors with different dielectric thicknesses. During the measurement process, $V_{DS}$ is fixed at 1 V.

## 4. High-frequency and low-power consumption properties

We developed a measurement system (as shown in Supplementary Figure 6a) to further explore the high-frequency properties of the demonstrated *AND* logic gate. Because of the limited sampling rate of source measurement unit (SMU), the output signal can only be read by oscilloscope. We adopted a current amplifier (Supplementary Figure 6a) to convert the output current signal into voltage signal. The high-frequency input signals were generated by pulse measurement unit (PMU) of Keithley 4200-SCS. Under 5 MHz and 2 MHz working frequency, the logic output signals of the *AND* logic gate are plotted in Supplementary Figure 6b and c (the yellow and green lines are the $V_{BG}$ and $V_{TG}$ input signals, red dotted lines are the output signals).

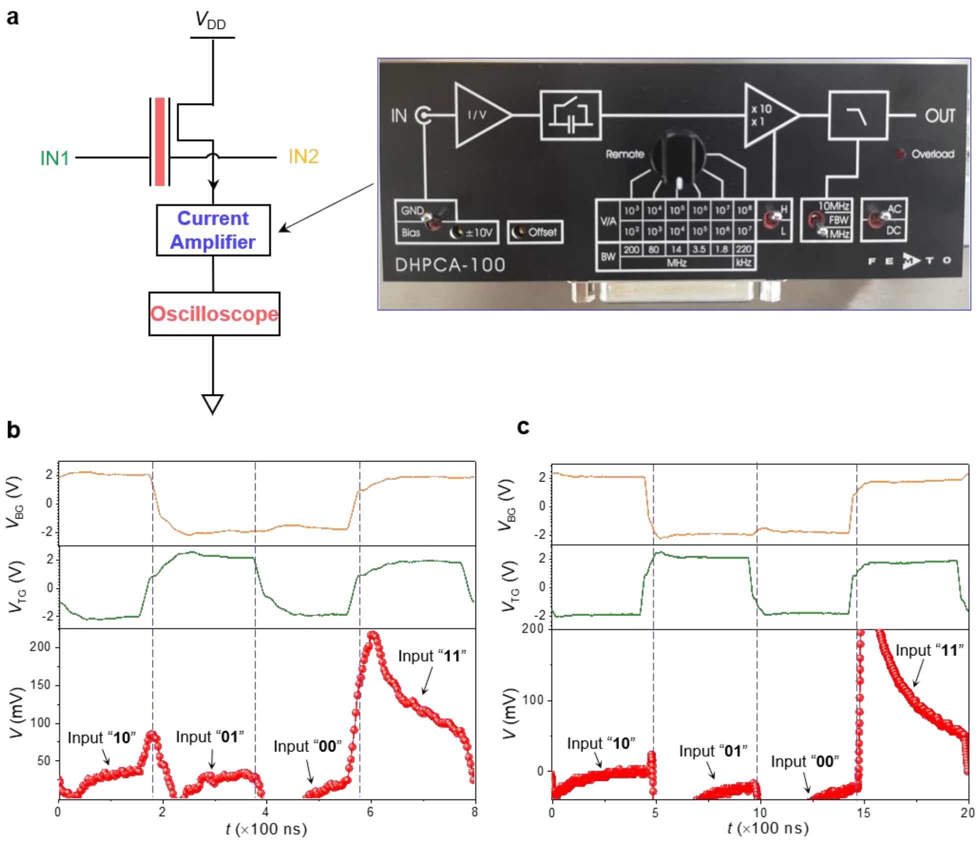


**Supplementary Figure 6.** High-frequency properties of the *AND* logic gate. **a**,

Circuit diagram of the high-frequency measurement system, the right part is the physical photo of the current amplifier. **b**, The logic output signal under 200-ns duration $V_{TG}$ input signal, which corresponds to 5 MHz. **c**, The logic output signal under 500-ns duration $V_{TG}$ input signal, which corresponds to 2 MHz.

Supplementary Figure 7 shows the gate leakage current ($I_G$) of the logic-memory transistor under different bottom and top gate voltage biases, which are essential for the subsequent power consumption calculation. The power consumption of the entire logic cycle can be described as:

$$E = P_{00}t_{00} + P_{01}t_{01} + P_{10}t_{10} + P_{11}t_{11} \qquad (1)$$

where $E$ is the power consumption sum of the 4 logic operations ("00", "01", "10" and "11"), $P$ represents the power, and $t$ is the duration of each logic operation. The power of the transistor during the logic operation is defined as:

$$P = V_{BG}I_{BG} + V_{TG}I_{TG} + V_{DS}I_{DS} \qquad (2)$$

where $V_{BG}$ and $V_{TG}$ are the bottom and top gate voltage during the logic operation; $I_{BG}$ and $I_{TG}$ are the bottom and top gate leakage current during the logic operation; $V_{DS}$ is the source-drain voltage bias and $I_{DS}$ is the channel current. Therefore, under 5 MHz working frequency (the duration of each logic operation is 200 nanoseconds), $E$ approximately equal to $4*10^{-14}$ J (40 fJ), which indicates that the power consumption of entire logic cycle is about 40 fJ.

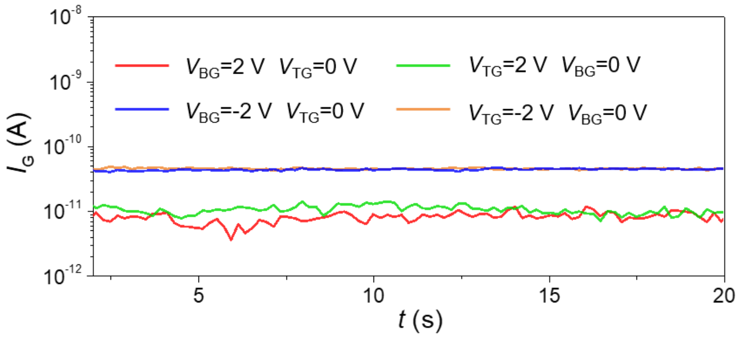


**Supplementary Figure 7.** Gate leakage current of the *AND* logic gate. Different lines represent the gate leakage current under different voltage biases.

When the logic gate does not perform logic operations (no input signals), in other word, the logic gate is in standby state, subthreshold leakage current ($I_{SUB}$) is the main source of standby power consumption.[4,5] Based on this consideration, the subthreshold leakage current of the logic-memory transistor under different source-drain voltage ($V_{DS}$) biases was measured, as shown in Supplementary Figure 8. When $V_{DS}$ is 1 V, the calculated standby power is around 40 pW.

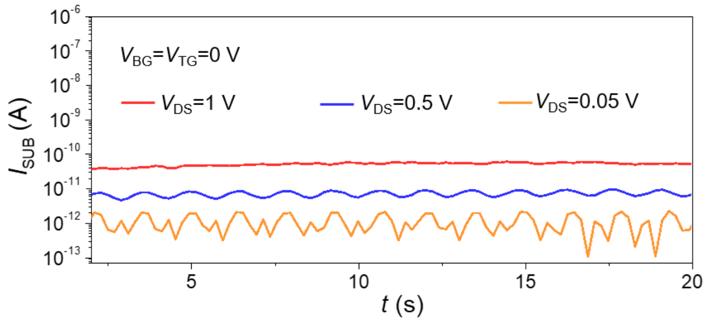


**Supplementary Figure 8.** Subthreshold leakage current of the logic-memory transistor. Yellow line, blue line and red line represents the $I_{SUB}$ under 0.05 V, 0.5 V and 1 V $V_{DS}$ bias respectively. During the measurement, $V_{BG}$ and $V_{TG}$ were kept at 0 V.

## 5. Implementation of *NAND* logic gate

In addition to *AND*, *NAND* is another important basic logic gate. By connecting a pull-up resistor (1 MΩ), we further realized *NAND* logic function on the constructed logic-memory transistor, the circuit diagram is shown in Supplementary Figure 9a. Supplementary Figure 9c presents the output signals under different input signals, which is corresponding to the truth table of *NAND* logic gate (Supplementary Figure 9b), only in the case of inputting “11”, logic output is “0”, which is opposite of *AND* logic gate. During the measurement, $V_{DD}$ is fixed at 1 V and the output voltage signal ($V_{OUT}$) is read by oscilloscope.

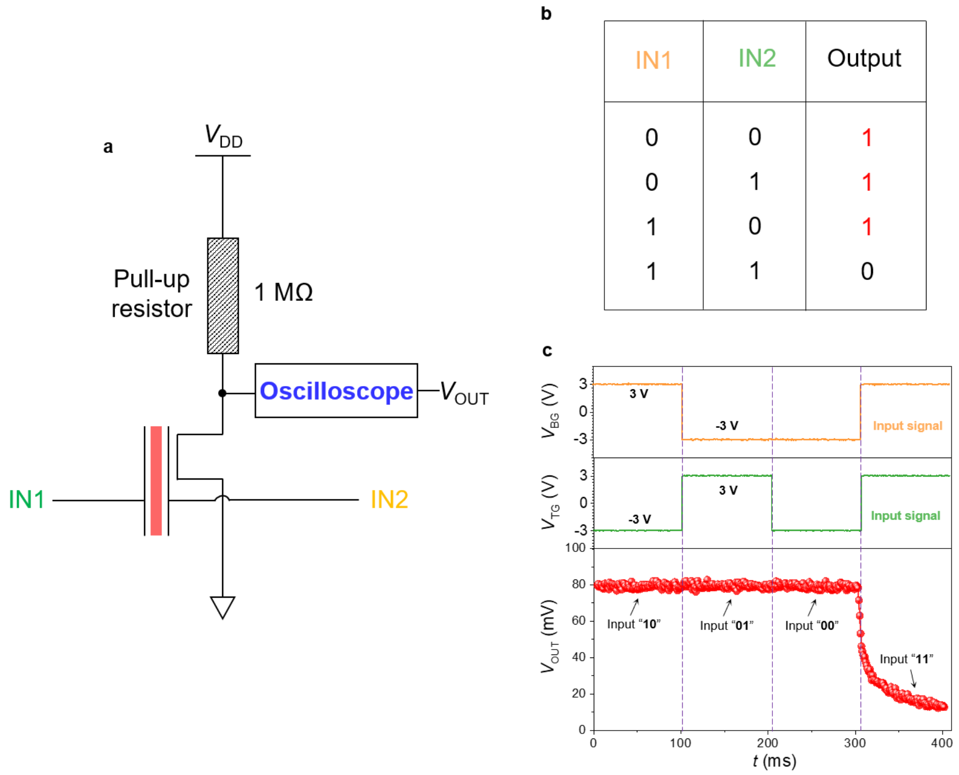


| IN1 | IN2 | Output |
|---|---|---|
| 0 | 0 | 1 |
| 0 | 1 | 1 |
| 1 | 0 | 1 |
| 1 | 1 | 0 |

**Supplementary Figure 9.** Implementation of *NAND* logic function on the constructed logic-memory transistor. **a**, Circuit diagram of the *NAND* logic function measurement system. **b**, The truth table of *NAND* logic function. **c**, *NAND* logic output signals.

## 6. Dynamic logic processing & *in situ* results storage properties

Supplementary Figure 10 demonstrates the dynamic response of the *AND* logic gate with *in situ* results storage capabilities under different input signals. The sequence of the input signals is listed as: "00" → "11" → "00" → "11" → "00" → "11", and there is a standby time between every two logic operations. As shown in Fig. 5a, in the standby time of the logic-memory transistor, matched with the last logic operation, the channel current value exhibits alternating high (ON) and low (OFF) changes.

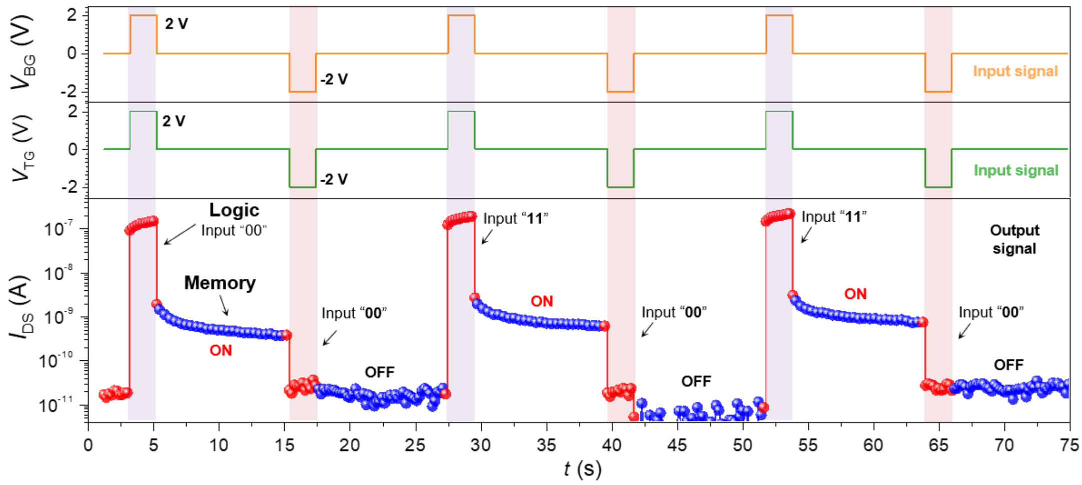


**Supplementary Figure 10.** Dynamic logic processing and *in situ* result storage properties of the logic-memory transistor. Respectively, the red and blue dotted lines represent the logic processing outputs and stored logic results. During the measurement, $V_{DD}$ is fixed at 1 V.

## 7. Mechanism of *in situ* results storage capabilities

To clarify the microscopic mechanism of the *in situ* logic results storage capabilities, top/bottom gate terminal regulation capability should be studied first. Therefore, we measured the transfer curves ($I_{DS}$-$V_{TG}$ and $I_{DS}$-$V_{BG}$) of the logic-memory transistor, as plotted in Supplementary Figure 11 and Supplementary Figure 12. When $V_{TG}$/$V_{BG}$ is swept form -5 V to +5 V (forward sweeping curve) and then from +5 V back to -5 V (reverse sweeping curve). Specifically, the threshold voltage ($V_{TH}$) of the reverse sweeping curve shows a negative shift compared to the $V_{TH}$ of the forward sweeping curve, which indicates that the direction of the $I_{DS}$-$V_{TG}$/$I_{DS}$-$V_{BG}$ sweeping curve is anticlockwise, and an obvious hysteresis window can be observed.

These phenomena are according with our expected theories: when the gate terminal is under a negative voltage bias, electrons can tunnel through the gate dielectric into the $WSe_2$ floating-gate; on the contrary, holes can tunnel through the gate dielectric into the $WSe_2$ floating-gate when the gate terminal is under positive voltage bias. The stored electrons in the floating-gate has a certain depletion effect on the $WSe_2$ channel, resulting in a positive shift of $V_{TH}$; conversely, the channel can be enhanced by the stored holes, resulting in a negative shift of $V_{TH}$.

We further draw the energy band diagrams and charge transport diagrams of the logic-memory transistor during different logic operations, as shown in Supplementary Figure 13. During the logic-00 operation, the negative gate voltage biases drive electrons tunnel into the floating-gate. After the logic operation, the stored electrons keep the channel in OFF state. After the logic-01 and logic-10 operations, the effects

produced by the stored holes and electrons will cancel each other out, therefore, the channel remains in OFF state. Meaningfully, After the logic-11 operation, the stored holes will generate a positive electric field, which maintains the output current at a relatively high value.

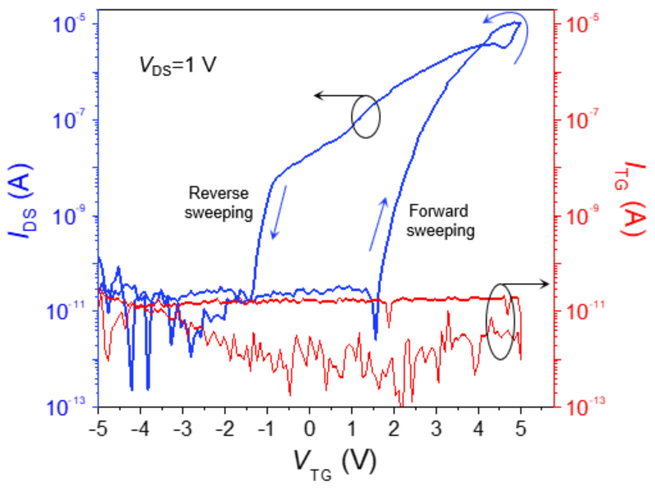


**Supplementary Figure 11.** Top gate modulated dual-sweeping transfer curve of the logic-memory transistor. Blue curve is the transfer curve, and red curve represents the top gate leakage current during the gate voltage sweeping process. $V_{DS}$ is 1 V

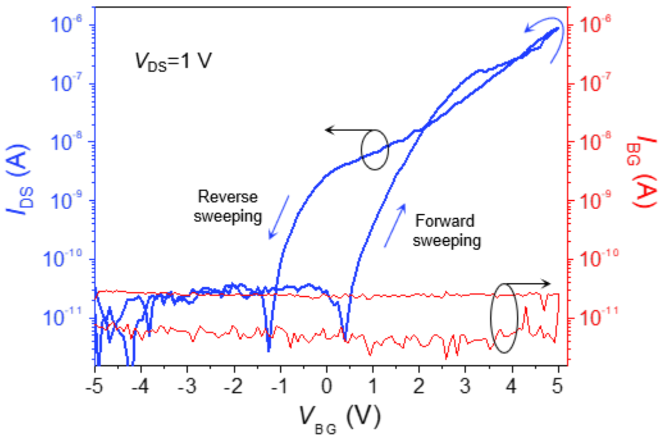


**Supplementary Figure 12.** Bottom gate modulated dual-sweeping transfer curve of the logic-memory transistor. Blue curve is the transfer curve, and red curve represents the bottom gate leakage current during the gate voltage sweeping process. $V_{DS}$ is 1 V.

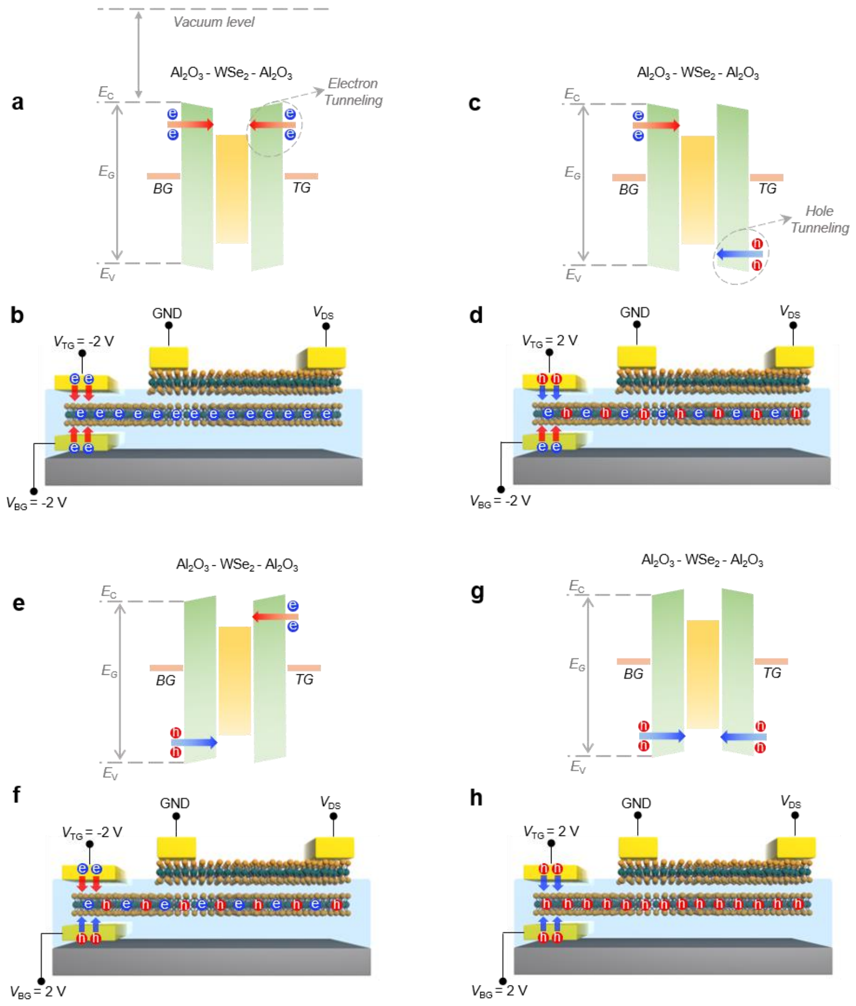


**Supplementary Figure 13.** Energy band diagrams and charge transport diagrams of the logic-memory transistor during the logic processing operations. **a**,**c**,**e**,**g**, the energy band diagrams of the logic-memory transistor under different gate voltage biases, specifically, $E_C$ and $E_V$ are the conduction band and valence band respectively, $E_G$ represents the band gap between $E_C$ and $E_V$. The red arrow and blue arrow indicate the electron tunneling and hole tunneling respectively. **b**,**d**,**f**,**h**, The charge tunneling processes during the logic operations, and charge storage in the floating-gate after the logic operations.

Based on the analyzation of the device operation mechanism, we would like to

point out that the relative positional relationship between the gate electrodes does not affect the realization of logic-memory functionalities. To support this conclusion, we give the measurement results of the logic-memory transistor that the two gate electrodes do not overlap each other, as shown in Supplementary Figure 14. Refer to the optical image of the device (Supplementary Figure 14a), it's clear that the bottom gate and top gate do not overlap with each other. The measurement results indicate that the device can perform stable *AND* logic function under 2 V and 3 V operation voltage range, as shown in Supplementary Figure 14b and c. Furthermore, as illustrated in Supplementary Figure 14d, the device can also execute logic-memory tasks.

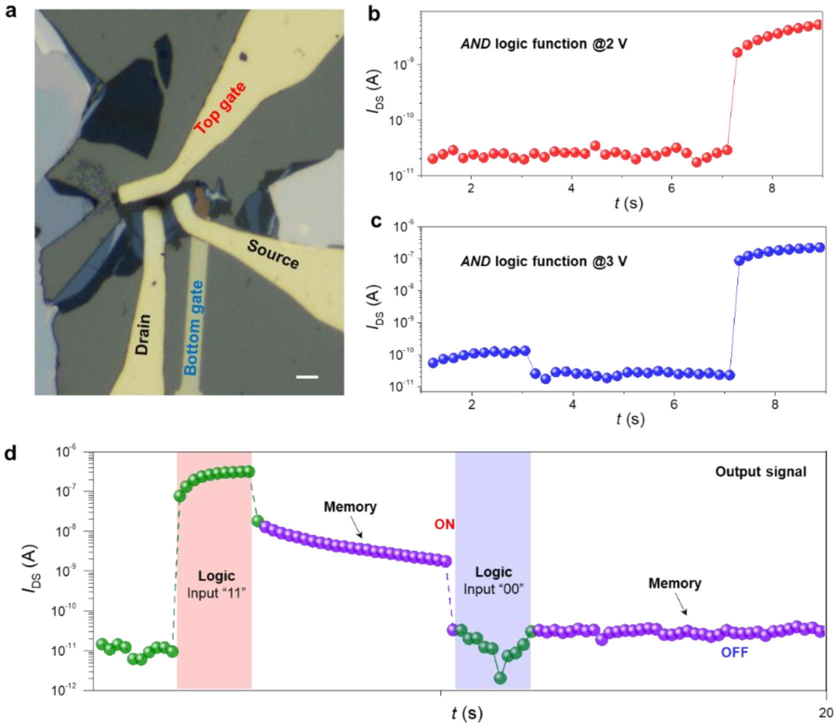


**Supplementary Figure 14. a**, the optical image of the device with two non-overlapping gate electrodes, the scale bar is 2 μm. **b**,**c**, the implementation of *AND* logic function under 2 V and 3 V operation voltage range. **d**, the dynamic logic-memory

characteristics of the device.

## 8. Optoelectrical and synaptic properties of the logic-memory transistor

Here, in order to further demonstrate the optoelectrical characteristics, we give the dynamic photoresponse of the logic-memory transistor. Supplementary Figure 15a demonstrates the dynamic photocurrent of the logic-memory transistor under the stimuli of interval blue laser pulses (the wavelength is 473 nm, the optical power density is 5.65 mW $cm^{-2}$ and the pulse width is about 900 ms). Obviously, following the switching of the laser signals, the photocurrent shows a regular rise and fall. In addition, we also applied one single blue laser pulse with long duration to the logic-memory transistor. As shown in Supplementary Figure 15b, in the beginning of the laser stimuli, the photocurrent shows a gradual increase process, then, the photocurrent reaches saturation. When the laser pulse is revoked, the photocurrent falls to the OFF state.

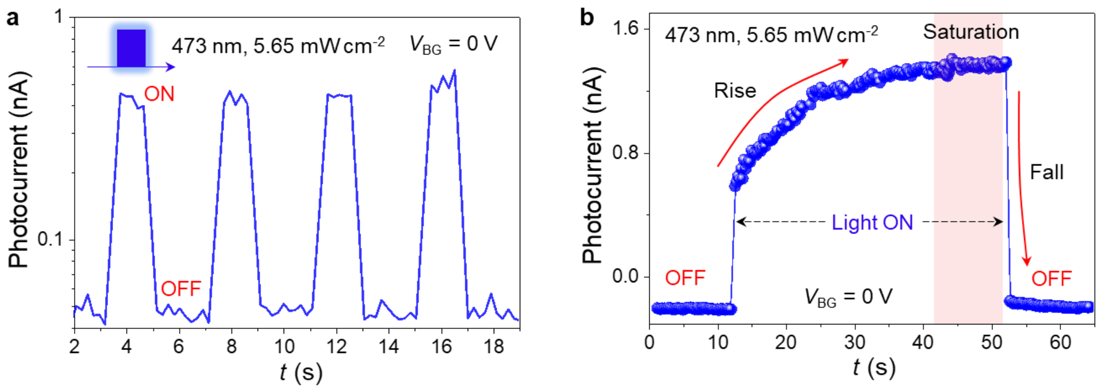


**Supplementary Figure 15.** Dynamic photoresponse of the logic-memory transistor. **a**, The measured photocurrent under the stimuli of interval laser pulses, during the measurement, the bottom gate voltage bias is 0 V and the drain-source voltage is fixed at 1 V. **b**, Photoresponse of the logic-memory transistor under the stimuli of single blue laser pulse.

Except the LTP and LTD synaptic behaviors, we also implemented paired-pulse facilitation (PPF) and spike-timing dependent plasticity (STDP) on the logic-memory transistor. Respectively, Supplementary Figure 16 and 17 presents the PPF and STDP properties of the logic-memory transistor.

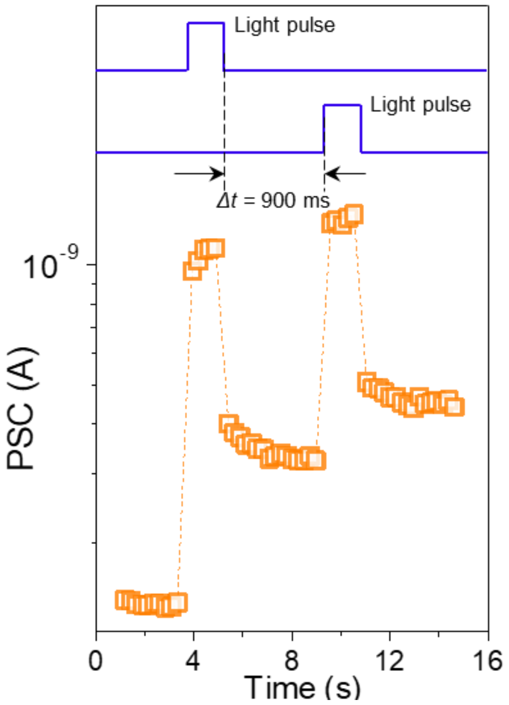


**Supplementary Figure 16.** The post-synaptic current of the device trigger by a pair of blue laser pulses (the wavelength is 473 nm and the optical power density is 5.65 mW cm$^{-2}$).

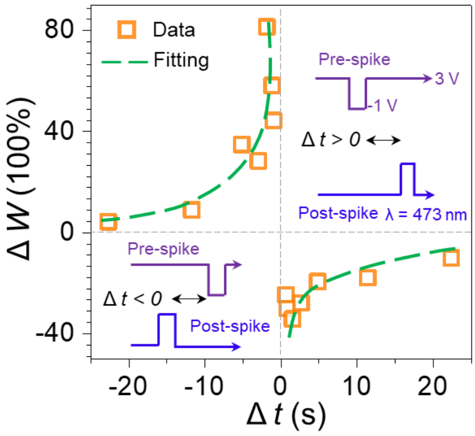

**Supplementary Figure 17.** The optical-electrical implementation of STDP on our fabricated device. During the measurement of STDP, electrical voltage pulses (with the pulse duration of 900 ms) are employed as pre-synaptic spikes, and optical blue laser pulses (the wavelength is 473 nm, the pulse width is 900 ms and the power intensity is 5.6 mW $cm^{-2}$) are the post-synaptic spikes. $\Delta W$ is the weight change (conductance change) of the synaptic device under the modulations of pre-spike and post-spike. $\Delta t$ is the relative timing between pre-spike signal and post-spike signal, ranging from -23 s to +23 s.

## 9. The optical images of the 3×3 logic-memory transistors

As we introduced in the manuscript, the proposed visible information sensing-memory-processing system is implemented on 3×3 logic-memory transistors. Noteworthily, during the image acquisition, memorization and distinction tasks, the data are all experimental measured. Here, we give the optical images of the 3×3 logic-memory transistors, as shown in Supplementary Figure 18.

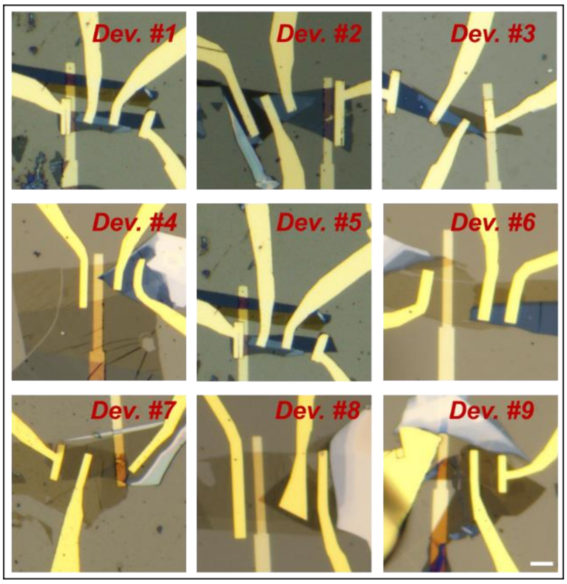


**Supplementary Figure 18.** The optical images of the 3×3 logic-memory transistors, the scale bar is 5 μm.

## 10. Calculation of the conductance change matrices

Here, we show more details about the calculation of the conductance change matrices during the letter image distinction processes. Take $M_1$ as an example, the second column of Supplementary Table 1 provides the detailed conductance value of the 3×3 logic-memory transistors in initial state 1, and the conductance values of the logic-memory transistors after the laser training of letter "Y" image are presented in the third column. $|\Delta C|$ is the conductance change absolute value of the logic-memory transistor before and after the training of letter "Y" image. $M_1$ is a third-order matrix, in which $M_{1mn}$ ($1\leq m, n\leq 3$) is equal to $\frac{|\Delta C|}{1^{-10}\ \mathrm{S}}$. Similar to $M_1$, the calculation of $U_1$, $M_2$ and $U_2$ are presented in Supplementary Table 2, 3 and 4, respectively.

| Cell number (m-n) | Initial 1 (S) | After training (S) | \|ΔC\| (S) | \|ΔC\|/(1E-10 S) |
|---|---|---|---|---|
| 1-1 | 6.26517E-11 | 4.27161E-10 | 3.64509E-10 | 3.65E+00 |
| 1-2 | 6.96773E-11 | 7.80576E-11 | 8.3803E-12 | 8.38E-02 |
| 1-3 | 7.47128E-11 | 3.28047E-10 | 2.53335E-10 | 2.53E+00 |
| 2-1 | 7.88963E-11 | 6.32969E-11 | 1.55994E-11 | 1.56E-01 |
| 2-2 | 8.48059E-11 | 3.15482E-10 | 2.30676E-10 | 2.31E+00 |
| 2-3 | 7.20381E-11 | 8.5916E-11 | 1.38779E-11 | 1.39E-01 |
| 3-1 | 7.62773E-11 | 7.21896E-11 | 4.08766E-12 | 4.09E-02 |
| 3-2 | 4.68561E-11 | 3.23506E-10 | 2.7665E-10 | 2.77E+00 |
| 3-3 | 8.526E-11 | 8.39479E-11 | 1.3121E-12 | 1.31E-02 |

**Supplementary Table 1.** The calculation of conductance change matrix $M_1$. The first column is the position number ($m$-$n$, $1\leq m, n\leq 3$) of the 3×3 logic-memory transistors.

| *Cell number (m-n)* | Before (S) | After (S) | \|ΔC\| (S) | \|ΔC\|/(1E-10 S) |
|---|---|---|---|---|
| *1-1* | 4.27161E-10 | 4.81865E-10 | 5.47042E-11 | 5.47E-01 |
| *1-2* | 7.80576E-11 | 7.19491E-11 | 6.1085E-12 | 6.11E-02 |
| *1-3* | 3.28047E-10 | 3.9234E-10 | 6.42925E-11 | 6.43E-01 |
| *2-1* | 6.32969E-11 | 7.53119E-11 | 1.2015E-11 | 1.20E-01 |
| *2-2* | 3.15482E-10 | 3.74324E-10 | 5.88423E-11 | 5.88E-01 |
| *2-3* | 8.5916E-11 | 8.6572E-11 | 6.56E-13 | 6.56E-03 |
| *3-1* | 7.21896E-11 | 2.1551E-10 | 1.43321E-10 | 1.43E+00 |
| *3-2* | 3.23506E-10 | 3.24414E-10 | 9.08357E-13 | 9.08E-03 |
| *3-3* | 8.39479E-11 | 2.07991E-10 | 1.24043E-10 | 1.24E+00 |

**Supplementary Table 2.** The calculation of conductance change matrix $U_1$. After inputting unknown letter 1 ("X") image onto the pre-trained "Y" template, the conductance of the nine logic-memory transistors is measured again, as presented in the third column of Supplementary Table 2. Similar to the calculation of matrix $M_{1,}$ $|\Delta C|$ is the conductance change absolute value of the logic-memory transistor before and after the inputting of unknown letter 1 ("X") image. $U_1$ is also a third-order matrix, in which $U_{1mn}$ ($1 \leq m, n \leq 3$) is equal to $\frac{|\Delta C|}{1^{-10}\ \mathrm{S}}$.

| *Cell number (m-n)* | Initial 2 (S) | After training (S) | \|ΔC\| (S) | \|ΔC\|/(1E-10 S) |
|---|---|---|---|---|
| *1-1* | 6.15415E-11 | 4.03493E-10 | 3.41951E-10 | 3.42E+00 |
| *1-2* | 7.73508E-11 | 7.89158E-11 | 1.565E-12 | 1.57E-02 |
| *1-3* | 5.89173E-11 | 3.19468E-10 | 2.60551E-10 | 2.61E+00 |
| *2-1* | 7.68264E-11 | 7.53624E-11 | 1.464E-12 | 1.46E-02 |
| *2-2* | 8.2434E-11 | 2.39582E-10 | 1.57148E-10 | 1.57E+00 |
| *2-3* | 6.32572E-11 | 6.69916E-11 | 3.73444E-12 | 3.73E-02 |
| *3-1* | 6.10873E-11 | 7.44101E-11 | 1.33228E-11 | 1.33E-01 |
| *3-2* | 5.48296E-11 | 3.6418E-10 | 3.09351E-10 | 3.09E+00 |
| *3-3* | 6.14405E-11 | 7.12308E-11 | 9.7903E-12 | 9.79E-02 |

**Supplementary Table 3.** The calculation of conductance change matrix $M_2$. After the distinction between letter "Y" image and unknown letter 1 ("X") image, we refreshed the 3×3 logic-memory transistors back to the initial state 2, and trained letter "Y" image again. The conductance of the nine logic-memory transistors before and after the training of letter "Y" image are presented in second and third column of Supplementary Table 3, respectively. As same as the calculation of matrix $M_{1,}$ $|\Delta C|$ is the conductance change absolute value of the logic-memory transistor before and after the training of letter "Y" image, and $M_{2mn}$ ($1\leq m, n\leq 3$) is equal to $\frac{|\Delta C|}{1^{-10}\text{ S}}$.

| *Cell number (m-n)* | Before (S) | After (S) | \|ΔC\| (S) | \|ΔC\|/(1E-10 S) |
|---|---|---|---|---|
| *1-1* | 4.03493E-10 | 4.68139E-10 | 6.46458E-11 | 6.46E-01 |
| *1-2* | 7.89158E-11 | 8.35603E-11 | 4.6445E-12 | 4.64E-02 |
| *1-3* | 3.19468E-10 | 3.85426E-10 | 6.59578E-11 | 6.60E-01 |
| *2-1* | 7.53624E-11 | 6.94559E-11 | 5.9065E-12 | 5.91E-02 |
| *2-2* | 2.39582E-10 | 2.9908E-10 | 5.94983E-11 | 5.95E-01 |
| *2-3* | 6.69916E-11 | 6.39133E-11 | 3.07834E-12 | 3.08E-02 |
| *3-1* | 7.44101E-11 | 6.08854E-11 | 1.35247E-11 | 1.35E-01 |
| *3-2* | 3.6418E-10 | 4.24083E-10 | 5.99021E-11 | 5.99E-01 |
| *3-3* | 7.12308E-11 | 8.62693E-11 | 1.50385E-11 | 1.50E-01 |

**Supplementary Table 4.** The calculation of conductance change matrix $U_2$. After inputting unknown letter 2 (“Y”) image onto the pre-trained “Y” template, the conductance change absolute value of the logic-memory transistor before and after the inputting of unknown letter 2 (“Y”) image is presented in the fourth column of Supplementary Table 4. $U_{2mn}$ ($1\leq m, n\leq 3$) is equal to $\frac{|\Delta C|}{1^{-10}\ \mathrm{S}}$.

## References


[1] Park, J. H. *et al.* Band structure engineering of layered $WSe_2$ *via* one-step chemical functionalization. *ACS Nano* (2019).

[2] Seo, S. *et al.* Direct *in situ* growth of centimeter- scale multi- heterojunction $MoS_2/WS_2/WSe_2$ thin- film catalyst for photo- electrochemical hydrogen evolution. *Adv. Sci.* 1900301 (2019).

[3] Muller, D. A. *et al.* The electronic structure at the atomic scale of ultrathin gate oxides. *Nature* **399**, 758 (1999).

[4] Roy, K., Mukhopadhyay, S. and Mahmoodi-Meimand, H. Leakage current mechanisms and leakage reduction techniques in deep-submicrometer CMOS circuits. *Proceedings of the IEEE* **91**, 305-327 (2003).

[5] Calhoun, B. H., Wang, A. and Chandrakasan, A. Modeling and sizing for minimum energy operation in subthreshold circuits. *IEEE J. Solid-State Circuits* **40**, 1778-1786 (2005).
